# Predictors of Symptomatic Intracranial Hemorrhage after Endovascular Thrombectomy in Acute Ischemic Stroke Patients with Anterior Large Vessel Occlusion—Procedure Time and Reperfusion Quality Determine

**DOI:** 10.3390/jcm11247433

**Published:** 2022-12-15

**Authors:** Yan Li, Natalie van Landeghem, Aydin Demircioglu, Martin Köhrmann, Philipp Dammann, Marvin Darkwah Oppong, Ramazan Jabbarli, Jens Matthias Theysohn, Jens-Christian Altenbernd, Hanna Styczen, Michael Forsting, Isabel Wanke, Benedikt Frank, Cornelius Deuschl

**Affiliations:** 1 Institute of Diagnostic and Interventional Radiology and Neuroradiology, University Hospital Essen, Hufelandstrasse 55, 45147 Essen, Germany; 2 Department of Neurology and Center for Translational Neuro- and Behavioral Sciences (C-TNBS), University Hospital Essen, Hufelandstrasse 55, 45147 Essen, Germany; 3 Department of Neurosurgery and Spine Surgery, University Hospital Essen, Hufelandstrasse 55, 45147 Essen, Germany; 4 Department of Radiology and Neuroradiology, Gemeinschaftskrankenhaus Herdecke, 58313 Herdecke, Germany; 5 Swiss Neuroradiology Institute, Bürglistrasse 29, 8002 Zürich, Switzerland

**Keywords:** brain, stroke, sICH, EVT, thrombectomy, LVO, brain hemorrhage

## Abstract

Purpose: We aimed to evaluate predictors of symptomatic intracranial hemorrhage (sICH) in acute ischemic stroke (AIS) patients following thrombectomy due to anterior large vessel occlusion (LVO). Methods: Data on stroke patients from January 2018 to December 2020 in a tertiary care centre were retrospectively analysed. sICH was defined as intracranial hemorrhage associated with a deterioration of at least four points in the National Institutes of Health Stroke Scale (NIHSS) score or hemorrhage leading to death. A smoothed ridge regression model was run to analyse the impact of 15 variables on their association with sICH. Results: Of the 174 patients (median age 77, 41.4% male), sICH was present in 18 patients. Short procedure time from groin puncture to reperfusion (per 10 min OR 1.24; 95% CI 1.071–1.435; *p* = 0.004) and complete reperfusion (TICI 3) (OR 0.035; 95% CI 0.003–0.378; *p* = 0.005) were significantly associated with a lower risk of sICH. On the contrary, successful reperfusion (TICI 3 and TICI 2b) was not associated with a lower risk of sICH (OR 0.508; 95% CI 0.131–1.975, *p* = 0.325). Neither the total time from symptom onset to reperfusion nor the intravenous thrombolysis was a predictor of sICH (per 10 min OR 1.0; 95% CI 0.998–1.001, *p* = 0.745) (OR 1.305; 95% CI 0.338–5.041, *p* = 0.697). Conclusion: Our findings addressed the paramount importance of short procedure time and complete reperfusion to minimize sICH risk. The total ischemic time from onset to reperfusion was not a predictor of sICH.

## 1. Introduction

Endovascular thrombectomy has become the standard therapy for selected acute ischemic stroke (AIS) patients due to anterior large vessel occlusion (LVO) because of its great efficacy in reducing patient disability at 90 days [1]. Despite recent advances in thrombectomy techniques and periprocedural management, intracranial hemorrhage (ICH) is a common complication, which was reported to be approximately 35% in a meta-analysis of 10,001 post-thrombectomy patients [2]. Symptomatic intracranial hemorrhage (sICH), which was tightly associated with poor neurological outcomes and high mortality, ranged from 4.4% (patients presenting with an early time window) to 7% (patients with an extended time window beyond 6 h) in randomized controlled trials [1,3,4]. In observational studies of stroke registries reflecting real-world practice, the reported rate of sICH differed from 4.4% to 16% under the circumstances of varying neurointerventional expertise and different definitions of sICH [5,6,7].

Understanding sICH predictors, especially modifiable ones, is of paramount importance to continuously improve the safety profile of the endovascular thrombectomy. Apart from established pre-procedural predictors such as hyperglycemia, high blood pressure and low baseline Alberta Stroke Programme Early CT Score (ASPECTS) [8], a long procedure time and unsuccessful reperfusion were among the strong and modifiable predictors for the occurrence of sICH [7,9,10]. However, recent meta-analysis indicated that complete reperfusion (TICI 3) should be pursued instead of successful reperfusion, which includes near-complete reperfusion (TICI 2b), as only complete reperfusion provided the highest safety profile with markedly lower rates of sICH and mortality [11]. Therefore, we conducted a retrospective analysis of our stroke data to corroborate this previously described association of reperfusion grade and the occurrence of sICH and to investigate predictive values of other pre- and peri-interventional factors for sICH. 

## 2. Materials and Methods

### 2.1. Patient Selection

**Figure 1:**
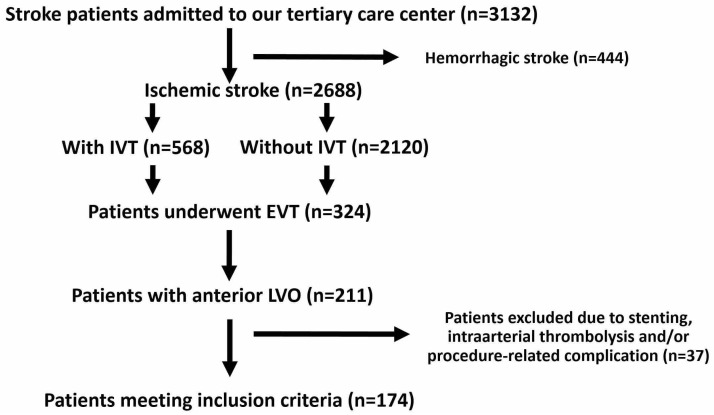
Flow chart of patient’s selection. Declaration of Abbreviations: IVT = intravenous thrombolysis; EVT = endovascular thrombectomy; LVO = large vessel occlusion.

This study was approved by the Ethics Committee of the University of Duisburg-Essen (number 19-9013-BO) and conducted in accordance with the Declaration of Helsinki. Informed consent was waived by the Ethics Committee. Prospectively registered stroke data from January 2018 to December 2020 from a tertiary care centre were retrospectively analysed. The eligible criteria for patients included: (a) acute ischemic stroke due to anterior LVO of the internal carotid artery and/or the M1/M2 branch of middle cerebral artery; (b) performed endovascular thrombectomy with aspiration catheter and/or stent retriever; (c) documented National Institutes of Health Stroke Scale (NIHSS) scores at hospital admission and discharge; (d) available CT control within 24 h after thrombectomy. Exclusion criteria were acute stenting of carotid artery and/or intracranial vessels, administration of intra-arterial thrombolytic agents and brain hemorrhage immediately detected on the intraprocedural angiogram due to procedure-related complications (e.g., vessel perforation or vessel injury by guidewire and/or stent). A detailed selection process with the patient’s number was summarized as flow chart (Figure 1). 

### 2.2. Clinical and Neurological Assessment

Timepoints of symptom onset and hospital admission were documented. In case of an unwitnessed stroke, the time of the last known well was defined as symptom onset. Beside age and gender, the pre-stroke modified Rankin Scale (mRS) and the disease history of coronary artery disease (CAD), diabetes mellitus (DM) and atrial fibrillation (AF) were evaluated. Neurological impairment was quantified with NIHSS scores and assessed at patient’s hospital admission and at 24/48/72 h intervals until discharge. 

### 2.3. Neuroimaging Analysis and In-Hospital Stroke Workflow 

All AIS patients underwent non-contrast CT followed by head–neck CT angiography for the detection of early ischemic changes and neurovascular occlusions immediately on arrival at the stroke centre. The extent of ischemic changes was classified with the ASPECTS score. Intravenous administration of a systemic thrombolytic agent with a recombinant tissue-type plasminogen activator was decided by the attending neurologist. Patients with severe neurological deficits due to emergent LVO were transferred directly to the angiosuite for endovascular recanalization. On patient arrival, the groin was punctured immediately during the preparation of general anaesthesia, which took an average of 15 min. The documented time from groin puncture to reperfusion included preparation and intubation time for general anaesthesia. Thrombectomy was performed using contact aspiration alone and/or a stent-retriever. Time points of groin puncture and final reperfusion were documented. The grade of reperfusion on the final angiogram was determined using the Thrombolysis in Cerebral Infarction Scale (TICI) [12]. First-pass recanalization was defined as TICI 3 after the first pass of the thrombectomy manoeuvre. Non-contrast CT was repeated 24 h after reperfusion therapy to reevaluate the extent of brain infarction and potential hemorrhage classified according to Heidelberger Bleeding Classification [13]. Intracranial hemorrhage that was associated with neurological deterioration of at least 4 NIHSS points compared to baseline or that led to death, which could not be explained by causes other than the observed ICH was defined as sICH [13]. 

### 2.4. Statistical Analysis

Patients were categorized into two groups: with sICH and without sICH. Baseline patient characteristics (age, gender, pre-stroke mRS, disease history, witnessed/unwitnessed stroke and NIHSS score at admission), separate time intervals from symptom onset to reperfusion (symptom onset to admission, door to groin and groin to reperfusion), intravenous thrombolysis (IVT), CT ASPECTS at baseline, TICI scores and first-pass effect were compared between the two groups. Mann–Whitney *U* test was run for comparison between numeric variables and χ^2^-test for nominal variables. 

A smoothed ridge regression model with logit-link was performed to estimate the effect of the variables on sICH [14]. The ridge regression was preferred over the more common logistic regression and step-wise feature selection, as it leads to more robust estimates given smaller sample sizes [15]. Multicollinearity was evaluated based on the variance inflation factor, with a factor >5 indicating multicollinearity between variables. Odds ratio (OR) with its 95% confidence interval (CI) was calculated to measure the association between each variable and the occurrence of sICH. To investigate the impact of different reperfusion grades on the occurrence of sICH, complete reperfusion (TICI 3) and successful reperfusion (TICI 2b/3) were tested in the regression model. The association of the total time from symptom onset to reperfusion with sICH was tested separately by replacing the different time intervals (symptom onset to admission, door to groin and groin to reperfusion) to avoid multicollinearity in the regression model.

SPSS (version 27, IBM, Armonk, NY, USA) was used for descriptive statistics. The smoothed ridge regression model was computed using R-Software (version 4.3, R Foundation for Statistical Computing, Vienna, Austria). A *p*-value < 0.05 was considered statistically significant.

## 3. Results

### 3.1. Baseline Characteristics and Neurological Outcome

A total of 174 patients met the inclusion criteria for analysis. Occlusion of ICA, M1 and M2 branch of middle cerebral artery were found in 35%, 43.7% and 21.2% of patients, respectively. TICI 3, TICI 2b and TICI 0–2a were achieved in 48.9%, 31% and 20.1% (TICI 0 = 14.9%, TICI 1 = 1.1% and TICI 2a = 4%) of patients, respectively. sICH was found in 10.3% (*n* = 18) of patients. A total of 33.3% (6/18) of sICH patients showed hemorrhagic infarction with confluent petechiae and another 22.2% (4/18) developed parenchymal hematoma occupying less than 30% of infarcted tissue. As the most common concomitant hemorrhage type, subarachnoid hemorrhage was found in seven patients. Incidence of hemorrhage types in the sICH is summarized in the Supplementary Materials. Patients with and without sICH did not differ regarding age, sex, previous disease history, pre-stroke mRS, witnessed/unwitnessed stroke, IVTrate, NIHSS at admission and ASPECTS scores (Table 1). However, the clinical outcome (NIHSS at discharge) was significantly worse in patients with sICH. 

### 3.2. Predictors of Symptomatic Intracranial Hemorrhage 

Analysis of variance inflation factors indicated no multicollinearities (variance inflation factors of all variables: <1.6). Compared to patients without sICH, the time intervals from groin to reperfusion were significantly longer (median time: 128 vs. 66.5 min, *p* < 0.001) and the percentage of complete perfusion was considerably lower in patients with sICH (5.6% vs. 53.8%, *p* < 0.001) (Table 1). In the smoothed ridge regression model, among the fifteen variables, short procedure time (per 10 min OR 1.24; 95% CI 1.071–1.435; *p* = 0.004) and complete reperfusion (TICI 3) (OR 0.035; 95% CI 0.003–0.378; *p* = 0.005) were significantly associated with the absence of sICH (Table 2). On the contrary, successful reperfusion, which included both TICI 3 and TICI 2b, did not remain a predictor for the absence of sICH (OR 0.508; 95% CI 0.131–1.975, *p* = 0.325). Total time from symptom onset to reperfusion was not a predictor of sICH (per 10 min OR 1.0; 95% CI 0.998–1.001, *p* = 0.745). 

### 3.3. Reperfusion Grade and Presence of Symptomatic Intracranial Hemorrhage

**Figure 2:**
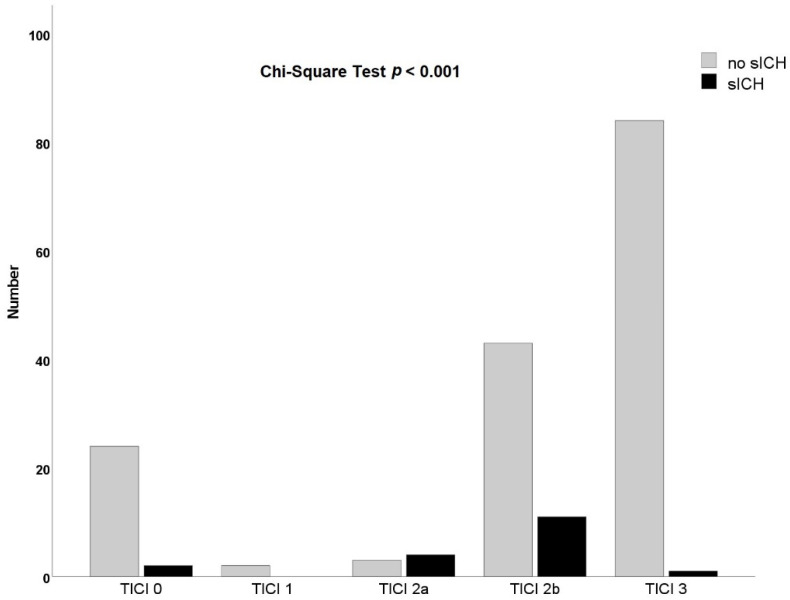
Reperfusion grade and presence of symptomatic intracranial hemorrhage. TICI = thrombolysis in cerebral infarction scale; sICH = symptomatic intracranial hemorrhage.

In patients who received complete reperfusion after thrombectomy, the prevalence of sICH was only 1.2% (1/85). Patients with partial reperfusion (TICI 2a) of occluded territory after thrombectomy showed the highest prevalence of sICH (57%, 4/7) followed by subtotal reperfusion (TICI 2b) (20.4%, 11/54) (Figure 2). With no reperfusion (TICI 0/1), the presence of sICH was 7% (2/28). Regarding different reperfusion grade, the prevalence of sICH was significantly different (*p* = 0.00000385, Chi-Square Test).

## 4. Discussion

In this retrospective analysis of 174 patients that underwent endovascular thrombectomy due to anterior LVO, our results revealed that complete reperfusion (TICI 3) and the short procedure time from groin to reperfusion were significantly associated with the absence of sICH. Neither the total ischemic time from stroke onset to reperfusion nor the IVT was a predictor of sICH. 

As demonstrated in previous studies, complete reperfusion was superior to near-complete reperfusion (TICI 2b) regarding patient’s early neurological improvement [16,17,18,19]. Moreover, rates of sICH and mortality were found to be notably lower in patients gaining complete reperfusion [11,19]. Our study confirmed the protective effect of complete reperfusion against the occurrence of sICH, since only 1 out of 85 patients developed sICH (1.2%). Similar results have been reported by other research groups who found an extremely low rate of sICH in 0.9% of patients experiencing complete reperfusion (1/113 by Darganzali et al.; 1/110 by Carvalho et al.) [17,20]. Consistent with previous work by Desai et al. [21] and regardless of the different definitions of sICH (SITS MOST, ECASS III and NINDS), our study revealed an inverse relation between reperfusion grade and incidence of sICH (Figure 2) with the highest rate of sICH in patients with TICI 2a. In contrast to TICI 3, patients achieving TICI 2b developed a much higher rate of sICH (20.4%). One explanation might be the fragmentation and distal migration of thrombi during the thrombectomy manoeuvre and the associated infarction, as well as the significantly increased rate of parenchymal hematoma [21,22]. Thus, successful reperfusion, including TICI 2b and TICI 3, did not remain a predictor for the absence of sICH (OR 0.508; 95% CI 0.131–1.975, *p* = 0.325), as the protective effect of TICI 3 was rather diluted by TICI 2b in our patient collective. To date, the association of successful reperfusion and the occurrence of sICH have been controversially discussed in the literature [7,10,22,23,24], although observational subgroup analyses and meta-analyses clearly strengthened the importance of pursuing complete reperfusion for a better neurological outcome and lower risk of sICH [11,19,22]. 

In our study, first-pass with TICI 3 could be reached in 28.2% of patients, which was very similar to the initial results (25.1% of patients) published by Zaidat et al. who first suggested the first-pass effect as an independent predictor of a good clinical outcome [25]. For the remaining patients without a first-pass effect, the question arose as to how many passes should be undertaken after the first pass in order to achieve a dedicated reperfusion grade, especially in the case of TICI 2b. Another approach to reach complete reperfusion should be weighed against the possibility of vessel injury and extended procedure time. As our data suggest and as previous studies have confirmed, a longer procedure time was independently associated with an increased rate of sICH [5,7,9]. Additionally, prolonged radiation exposure, extended anesthesia time and hemodynamic changes under sedation are important factors that influence decision making before further passes. Besides the safety issue, the odds of a good clinical outcome and the rate of complete recanalization declined progressively with each additional pass, although the detrimental effects of each additional pass may be counterbalanced by the benefits of a complete recanalization for up to three passes [26,27]. Based on our findings, attention should be given particularly in hyperacute intracranial atherosclerotic disease-related LVO. As more passes are needed, the procedure time may increase, often resulting in a lower final reperfusion grade as a consequence of early intraprocedural restenosis/reocclusion [28]. 

Apart from procedure time, our data suggested that neither the pre-procedural time (symptom onset to admission or door to groin puncture) nor the total ischemia time (symptom onset to reperfusion) was associated with sICH risk. To date, there are only limited and discrepant data regarding the impact of EVT time metrics on sICH occurrence. In a recently published prospective observational study, the rate of parenchymal hematoma increased by 2.5% (95% CI 1.5–3.6%, *p* < 0.001) for every additional hour of onset to reperfusion delay in patients with successful reperfusion (TICI2b-3) [29]. On the contrary, in two studies that compared the neurological outcomes of patient groups treated with drip-and-ship paradigm and in mothership site, the longer onset to reperfusion time of the drip-and-ship group was not associated with higher risk of overall intracranial hemorrhage or sICH [30,31]. Our results also confirmed that the longer ischemia time was not necessarily associated with a higher sICH risk.

The safety of IVT regarding sICH risk still remains a matter of debate. Compared to EVT alone, IVT treatment along with EVT was reported to be associated with higher risk of sICH (6.5% (685 of 10,530 patients) vs. 5.3% (279 of 5249 patients); OR, 1.28; 95% CI, 1.16–1.42; *p* < 0.001) in patients within 6 h of time last known well [32]. However, previous studies also suggested that the extent of early ischemia (measured with ASPECTS) might influence relative risks of sICH in IVT + EVT vs. EVT alone [33]. In our study, the IVT was not significantly associated with higher risk of sICH (OR 1.305; 95% CI 0.338–5.041, *p* = 0.697), probably due to little early ischemic change of the patients at the time of admission (mean ASPECTS = 10). 

Our study had several limitations. Firstly, there was selection bias. With a median time of 176 min from symptom onset to admission, the baseline CT of our patients showed often little or no early ischemic change. Patients with a large infarct demarcation on baseline CT, e.g., with lower ASPECTS < 7, were mostly excluded from endovascular reperfusion therapy. This selection bias may explain the missing correlation between ASPECTS and the occurrence of sICH, which has been repeatedly reported in previous studies [6,21,23]. Secondly, our sample size was limited, with a small number of sICH. Thirdly, we did not investigate the influence of the total number of thrombectomy passes, pre-treatment collateral scores and the different interventional experience of individual operators on the incidence of sICH. Finally, other known pre-procedural predictors such as blood glucose level or blood pressure [8] were not investigated. 

## 5. Conclusions

Our results demonstrated the upmost importance of a short procedural time and complete reperfusion to minimize the risk of post-thrombectomy sICH in AIS patients due to anterior LVO. Successful reperfusion, including TICI 2b and TICI 3, was not a predictor for the absence of sICH. Longer ischemia time from onset to reperfusion and IVT were not associated with a higher risk of sICH. Further prospective clinical trials with larger sample sizes are warranted to validate the protective effect of complete reperfusion in reducing the risk of sICH. 

## Figures and Tables

**Table 1:** Baseline characteristics, endovascular and neurological outcomes of patients.

Variables	All (*n* = 174)	Non-sICH (*n* = 156)	sICH (*n* = 18)	*p*-Values
**Age** (**years**) (**IQR**)	77 (67–84)	76.5 (67–84)	79 (54.5–81.25)	0.882
**Female** (**%**)	102 (58.6%)	91 (58.3%)	11 (61.1%)	0.821
**CAD** (**%**)	44 (25.3%)	40 (25.6%)	4 (22.2%)	0.752
**DM** (**%**)	47 (27%)	43 (27.6%)	4 (22.2%)	0.629
**AF** (**%**)	104 (59.8%)	92 (59%)	12 (66.7%)	0.529
**Pre-stroke mRS** (**median, IQR**)	1 (0–2)	1 (0–2)	1 (0–2.25)	0.990
**Witnessed Stroke** (**%**)	94 (54%)	85 (54.5%)	9 (50%)	0.718
**NIHSS at Admission** (**median, IQR**)	15 (9.75–20)	15 (9.25–20)	14 (10.5–20.5)	0.945
**NIHSS at Discharge** (**median, IQR**)	9 (3–42)	8 (3–27.25)	42 (11.75–42)	**0.001**
**Intravenous Systemic Thrombolysis** (**%**)	111 (63.8%)	99 (63.5%)	12 (66.7%)	0.789
**ASPECTS Score** (**median, IQR**)	10 (9–10)	10 (9–10)	10 (9–10)	0.522
**Symptom onset to Admission** (**median, IQR**)	176 (57–519)	172.5 (28–484)	253.5 (55.75–693.5)	0.656
**Door to Groin** (**median, IQR**)	66 (42–83)	66 (38.5–83)	64.5 (53.75–79.5)	0.882
**Groin to Reperfusion** (**median, IQR**)	70 (43–108)	66.5 (40–104)	128 (77.75–144.25)	**0.0003**
**TICI 3** (**%**)	85 (48.9%)	84 (53.8%)	1 (5.6%)	**0.0001**
**TICI 2b/3** (**%**)	139 (79.9%)	127 (81.4%)	12 (66.7%)	0.14
**First Pass Recanalization** (**%**)	49 (28.2%)	48 (30.8)	1 (5.6%)	0.071

CAD = coronary artery disease; DM = diabetes mellitus; AF = atrial fibrillation; mRS = modified Rankin scale; NIHSS = National Institutes of Health Stroke Scale; ASPECTS = Alberta Stroke Program early CT Score; TICI = thrombolysis in cerebral infarction scale; IQR = interquartile range; sICH = symptomatic intracranial hemorrhage. Significant *p*-values are marked in bold.

**Table 2:** Predictors of symptomatic intracranial hemorrhage in a smoothed ridge regression model.

Variables	OR	CI (95%)	*p*-Values
**Age** (**per year**)	1.005	0.954–1.058	0.852
**Female**	1.635	0.415–6.443	0.479
**CAD**	0.703	0.153–3.226	0.648
**DM**	0.636	0.149–2.709	0.537
**AF**	2.245	0.597–8.441	0.228
**Pre-stroke mRS**	1.099	0.671–1.8	0.705
**Witnessed Stroke**	0.621	0.118–3.268	0.571
**NIHSS at Admission**	0.971	0.893–1.055	0.478
**Intravenous Thrombolysis**	1.305	0.338–5.041	0.697
**ASPECTS Score**	1.44	0.715–2.898	0.303
**Symptom onset to Admission** (**per 10 min**)	0.996	0.978–1.014	0.65
**Door to Groin** (**per 10 min**)	1.033	0.884–1.207	0.678
**Groin to Reperfusion** (**per 10 min**)	1.24	1.071–1.435	**0.004**
**TICI 3**	0.035	0.003–0.378	**0.005**
**TICI 2b/3**	0.508	0.131–1.975	0.325
**First Pass Effect**	4.402	0.496–39.094	0.18

CAD = coronary artery disease; DM = diabetes mellitus; AF = atrial fibrillation; mRS = modified Rankin scale; NIHSS = National Institutes of Health Stroke Scale; ASPECTS = Alberta Stroke Program early CT Score; TICI = thrombolysis in cerebral infarction scale; sICH = symptomatic intracranial hemorrhage. Significant *p*-values are marked in bold. OR = Odds Ratio; CI = confidence interval.

## Data Availability

The data presented in this study are available on request from the corresponding author.

## Supplementary Materials

The following supporting information can be downloaded at: https://www.mdpi.com/article/10.3390/jcm11247433/s1, Table S1: Incidence of hemorrhage types according to Heidelberger Bleeding Classification in patients with symptomatic intracranial hemorrhage.
