# Supplementary material for: Predictors of Symptomatic Intracranial Hemorrhage after Endovascular Thrombectomy in Acute Ischemic Stroke Patients with Anterior Large Vessel Occlusion—Procedure Time and Reperfusion Quality Determine
Source: J Clin Med. 2022 Dec 15;11(24):7433. doi: 10.3390/jcm11247433 (PMC9781417; doi:10.3390/jcm11247433)
Supplement: Supplementary file 1 [file jcm-11-07433-s001.zip › jcm-2068518-supplementary.pdf]

Title: Predictors of symptomatic intracranial hemorrhage after endovascular thrombectomy in acute ischemic stroke patients with anterior large vessel occlusion – procedure time and reperfusion quality determine

Supplementary Material

Table S1. Incidence of hemorrhage types according to Heidelberger Bleeding Classification in patients with symptomatic intracranial hemorrhage

	**H1**	**H2**	**PH1**	**PH2**	**3a**	**3b**	**3c**	**3d**
**Symptomatic ICH (n = 18)**	0	6 (33.3%)	4 (22.2%)	1 (5.6%)	3 (16.7%)	2 (11.1%)	7 (38.9%)	1 (5.6%)

Declaration of Abbrevations: H1) Scattered small petechiae, no mass effect; H2) Confluent petechiae, no mass effect; PH1) Hematoma within infarcted tissue, occupying <30%, no substantive mass effect; PH2) Hematoma occupying 30% or more of the infarcted tissue, with obvious mass effect; 3a) Parenchymal hematoma remote from infarcted brain tissue; 3b) Intraventricular hemorrhage; 3c) Subarachnoid hemorrhage; 3d) Subdural hemorrhage
